# The Canadian Network for Research in Schizophrenia and Psychoses: A Nationally Focused Approach to Psychosis and Schizophrenia Research

**DOI:** 10.1177/07067437211009122

**Published:** 2021-04-19

**Authors:** Tania Lecomte, Jean Addington, Chris Bowie, Martin Lepage, Stéphane Potvin, Jai Shah, Chris Summerville, Phil Tibbo

**Affiliations:** 1 Department of Psychology, University of Montreal, Quebec, Canada; 2 Department of Psychiatry, University of Calgary, Alberta, Canada; 3 Department of Psychology, Queen’s University, Kingston, Ontario, Canada; 4 Department of Psychiatry, McGill University, Montreal, Quebec, Canada; 5 Department of Psychiatry and Addictology, University of Montreal, Quebec, Canada; 6 Schizophrenia Society of Canada, Steinbach, Manitoba, Canada; 7 Department of Psychiatry, University of Dalhousie, Halifax, Nova Scotia, Canada

**Keywords:** schizophrenia, research, network

Schizophrenia and other psychotic disorders affect approximately 4% of the population (more than 1.5 million Canadians) and indirectly affect many more family members, friends, and other supporters.^1,2^ Schizophrenia rates among the world’s top 10 causes of disability-adjusted life-years,^3^ with most psychosocial deterioration in schizophrenia occurring in the first 5 years of onset, if not sooner. Since the age of onset of psychosis occurs in late adolescence or early adult years, it has the potential to negatively affect the lives of many young Canadians, during a developmental window when they are developing the life skills and experiences needed for independent living. Due to the potential severity of the illness and chronicity starting at an early age, schizophrenia and other psychotic disorders have significant direct and indirect costs not only in terms of treatment but also related to unemployment, suicide (5% rate),^4^ physical illnesses, and overall reduced life expectancy (in 2004 alone, 374 Canadians died prematurely from schizophrenia).^5^ In Canada, the cost of schizophrenia has been estimated to be up to CAD$10 billion CA annually (from CAD$7 billion in 2004).^5^ Ultimately, the personal, family, and societal cost/burden of schizophrenia is significant. A Canadian report on the hypothetical impact of affecting the causes of schizophrenia by as little as 10% (e.g., by targeting obstetric complications or environmental risk factors such as cannabis use), as well as improving remission by only 10% (by improving protective factors via treatment),^6^ suggested that more than 12,000 Canadians who would otherwise have schizophrenia would be illness-free within less than 10 years.^7^ Such impacts can only be achieved with quality Canadian research in the field.

There have been substantial advancements in our understanding of schizophrenia and its treatment that have contributed to improved long-term outcomes. The role of early intervention services (EIS) for psychosis, for instance, has been well-documented to improve long-term outcomes and while EIS in Canada has been developed based on local resources (without a national strategy and funding), there is some degree of consistency across Canadian programs in service delivery.^8^ To note, EIS for psychosis in Canada now has a national organization that is focused on knowledge translation, the Canadian Consortium for Early Intervention in Psychosis (CCEIP: www.epicanada.org) which reflects Canada’s long history of innovation and leadership in this area.

International experts in the field of schizophrenia published a report, endorsed by a number of leading international organizations, addressing current needs in schizophrenia care policies (*Schizophrenia: Time to Commit to Policy Change*).^9^ Recommendations for policy change/action areas to focus included (1) provision and support of evidence-based integrated care, (2) support for entry/re-entry into vocational and educational programs, (3) support and education for families, (4) engagement of all stakeholders on a regular basis to update policies on the management of schizophrenia, and (5) encourage funded awareness campaigns to aid in the reduction of stigma. What is interesting in this report with respect to research is that it clearly advocates to “Provide support, which is proportionate to the impact of the disease, for research and development of new treatments that improve the overall outlook for people with schizophrenia, including those that target negative symptoms and cognitive impairment.”

In Canada, in 2017, experts in the field using strict guidelines and reviewing methodologies developed the Canadian Guidelines for Treatment of Schizophrenia and Psychoses.^10^ These best practices represented state-of-the-art knowledge regarding evidence-based care. Nonetheless, many questions remain unanswered with respect to improving the lives of people with schizophrenia and psychosis and their families. There remains a strong need for continued research into most research domains from the biological underpinnings of schizophrenia and its treatment to recovery-promoting services and policy implementation.

This opportunity to evaluate the potential for additional best practices is, unfortunately, set against a backdrop of years of decreases in health research funds in Canada. Indeed, Canada currently spends 1.54% of its gross domestic spending on research and development (all domains together), which is much lower than the 4.8% spent by Israel or even the 2.8% spent by the United States.^11^ Although health funding has increased recently, previous cuts have resulted in current investment in health research being similar to that of 10 years ago^12^ Of course, even fewer funds are available for schizophrenia and psychosis research. For example, last year, Canadian Institutes of Health Research (CIHR) gave out CAD$253 million in Canadian funds (excluding COVID funds): 6 of 336 funded projects (1.8%) pertained to, or at least included people with, schizophrenia or psychosis (total of CAD$3.7 million). As a comparison, 54 proposals on cardiovascular problems were funded (16%, total of CAD$33.8 million), whereas cardiovascular problems cost CA CAD$22 billion annually, only twice the cost of schizophrenia. Should the funding be proportional to the cost of the disease (as suggested above in the international report),^9^ CAD$15 million should be invested in research for schizophrenia or psychosis, which is 5 times the actual amount invested. Although it is difficult to estimate the actual percentage of funded proposals in this field, it is clear that more research (and perhaps more researchers) are needed in schizophrenia and psychosis research. Some might argue that mental health has always been the poor parent in terms of federal and provincial health funding. Yet, funding research in schizophrenia holds the potential to significantly diminish the direct and indirect costs linked to the illness by decreasing relapse rates, helping sustained remission, improving citizenship and social recovery, diminishing stigma, and preventing the emergence of the symptoms and deficits linked to the disorder.

Canada is home to many strong and internationally recognized researchers in schizophrenia and psychosis. These experts include junior to senior investigators, spanning the domains of brain research, genetics, and psychosocial determinants to treatment, implementation, and policies. When times are hard, there is a need to join forces and to prioritize what should be funded. As such, we wish to present the recently created Canadian Network for Research in Schizophrenia and Psychoses (CNRSP).

## Why the Need for a National Structure for Schizophrenia Research in Canada?

A national network can be more responsive to Canadian needs by helping target research priorities and advocate for more funding for research in schizophrenia and psychosis. It can also foster partnering and collaborations, thus developing a community of research. The Canadian Network for Mood and Anxiety Treatments (canmat.org), for instance, has helped increase communication between researchers and clinicians working in anxiety and depression, enabling the development of clinical guidelines and more concerted research. The Canadian Sleep and Circadian Network (cscnweb.ca) not only created novel studies (biological and genetic) and a data bank but also led to a national education campaign on sleep disorders. The Canadian Consortium on Neurodegeneration in Aging (ccna-ccnv.ca) has significantly accelerated the development of research collaborations and national platforms to support research in key areas including prevention, treatment, and quality of life. The CIHR-funded Canadian Longitudinal Study on Aging represents a complementary example on how a strategic funding initiative can help develop research capacity in a critical area of health research by generating a unique data platform that is following more than 50,000 Canadians for a 20-year period. This ambitious project has received more than CAD$100 million in Canadian funding since its inception in 2001 and a new phase of funding for up to CAD$52.2 million is projected.

Researchers in schizophrenia and psychosis can increase the impact of Canadian studies in schizophrenia and psychosis by coming together, developing, and using technologies to increase, for example, pooled data, shared measures, or multiple recruitment sites. Larger samples can be collected across the country and enable greater generalization of the results. Sharing of expensive or difficult-to-collect data (such as imaging or genetics) can also help increase the impact of Canadian studies and help improve the lives of people affected. A Canadian network can maximize the training of fellows, graduate students, post-docs, and emerging researchers by facilitating collaboration, exchanges, and lab visits across Canada. Overall, a Canadian network can make unique contributions to improve the health of Canadians at risk of developing, or who currently have, schizophrenia or a psychotic disorder.

## Development of the CNRSP

In 2019, the current director of the Network (Tania Lecomte) reached out to fellow researchers in schizophrenia/psychosis to verify whether others were facing challenges due to a lack of funding and to gauge the level of interest in joining forces to promote research in this field. Within 2 weeks, over 50 researchers from across the country had joined the Network. The first online meeting helped decide our mission statement and vision.

The CNRSP would offer the structure for the coordination of schizophrenia research nationally, with the following vision—“Recovery is possible for people with schizophrenia and psychosis”—and the mission:

- *To raise awareness that continued research in schizophrenia and psychotic disorders is needed and greatly contributes to improving the recovery and quality of lives of people affected (individuals and their families), with a resulting decrease in health and social costs related to these disorders.*
- *To improve the impact of Canadian schizophrenia research by fostering interdisciplinary collaborative efforts and creating momentum to generate resources needed to answer current gaps and needs as determined by multiple stakeholders including researchers, clinicians, families, and people with lived experience.*
- *To acknowledge the importance, and further expansion, of policy development and knowledge translation, integral to Canadian research efforts in schizophrenia and psychosis.*

It was decided that stakeholders believing in the value of research in schizophrenia, but who did not hold formal independent investigator positions, should nonetheless be invited to join the Network. As such, the Schizophrenia Society of Canada, the CCEIP, clinicians, family members, trainees, people with lived experience, and policy-makers were all invited to play an active role in the Network. Our first step was to help funding agencies target essential domains in research. To do this, we reached out to psychosis and schizophrenia clinicians and researchers, mental health organizations, individuals with lived experience, and family members/carers in Canada to survey the current needs and priorities for schizophrenia research in Canada. It is essential that Canadian research be driven by current needs as described by the individuals themselves and their families as well as by gaps in knowledge in order to help prevent the development of these disorders. Over 200 surveys were received with responses thematically categorized by research staff into 4 general areas: (1) need for increased service delivery research (e.g., quality, fidelity), (2) need for investigations around novel treatments (pharmacological and non-pharmacological), (3) need for expanded qualitative and psychosocial research (e.g., stigma, recovery, families, care teams), and (4) additional neurobiological studies on etiology and prevention.

A face to face national meeting of clinicians and researchers, individuals representing mental health organizations, policy-makers, carers, and individuals with lived experience took place on January 24, 2020, in Montreal. At this meeting, the organizational committee (authors of this article) chaired discussions on the 4 areas that emerged from the survey to allow for a clear and thorough understanding of the needs and ultimately the approach to schizophrenia research in Canada. This discussion led to the emergence of several main subthemes for schizophrenia and psychosis research which should be targeted in the near future by Canadian researchers. These included but are not limited to:

- the use of digital technologies for research and their investigation for use in service delivery,
- improvement of knowledge and use of biomarkers in schizophrenia research,
- standardization of measurement-based care and service delivery models across the life trajectory (including implementation science),
- how to improve life expectancy and physical health, and
- understanding and overcoming barriers to recovery including substance misuse and resistance to treatment.

As a result of this meeting, The CNRSP, aligned with existing stakeholders and proposing to work within a recovery model, will strive to improve care, services, and understanding of psychosis and schizophrenia through research at the national level. Members of the Network have volunteered for subcommittees, focusing on lobbying, funding, knowledge transfer, and education/stigma reduction.

The *CNRSP* has already started creating bridges and facilitating research collaborations nationally. We will strive to answer the needs of people with schizophrenia and psychosis via our studies in order to decrease the personal, family, and societal burden of these disorders. More information about joining the Network can be obtained by contacting us or via our website: www.schizophrenianetwork.com.
